# DECIPHER: database for the interpretation of phenotype-linked plausibly pathogenic sequence and copy-number variation

**DOI:** 10.1093/nar/gkt937

**Published:** 2013-10-21

**Authors:** Eugene Bragin, Eleni A. Chatzimichali, Caroline F. Wright, Matthew E. Hurles, Helen V. Firth, A. Paul Bevan, G. Jawahar Swaminathan

**Affiliations:** ^1^Wellcome Trust Sanger Institute, Wellcome Trust Genome Campus, Hinxton, Cambridge CB10 1SD, UK and ^2^Cambridge University Department of Medical Genetics, Addenbrooke’s Hospital, Cambridge CB2 2QQ, UK

## Abstract

The DECIPHER database (https://decipher.sanger.ac.uk/) is an accessible online repository of genetic variation with associated phenotypes that facilitates the identification and interpretation of pathogenic genetic variation in patients with rare disorders. Contributing to DECIPHER is an international consortium of >200 academic clinical centres of genetic medicine and ≥1600 clinical geneticists and diagnostic laboratory scientists. Information integrated from a variety of bioinformatics resources, coupled with visualization tools, provides a comprehensive set of tools to identify other patients with similar genotype–phenotype characteristics and highlights potentially pathogenic genes. In a significant development, we have extended DECIPHER from a database of just copy-number variants to allow upload, annotation and analysis of sequence variants such as single nucleotide variants (SNVs) and *InDels*. Other notable developments in DECIPHER include a purpose-built, customizable and interactive genome browser to aid combined visualization and interpretation of sequence and copy-number variation against informative datasets of pathogenic and population variation. We have also introduced several new features to our deposition and analysis interface. This article provides an update to the DECIPHER database, an earlier instance of which has been described elsewhere [Swaminathan *et al.* (2012) DECIPHER: web-based, community resource for clinical interpretation of rare variants in developmental disorders. *Hum. Mol. Genet.*, 21, R37–R44].

## INTRODUCTION TO DECIPHER

Genetic diseases are caused by abnormalities in the genome of an individual that range from small mutations in critical genes, to larger structural changes that include deletions, duplications, translocations or inversions in regions of chromosomes (or combinations of the above) and affect one or more genes. The external manifestation of these disorders (phenotype) in combination with genetic testing to determine the underlying cause (genotype) is usually necessary for accurate clinical diagnosis for these rare disorders. For some genetic disorders, while phenotype assessments alone may provide strong indicators to an underlying condition [Down syndrome, (2)], genetic assessment is still essential to confirm the genetic basis of a phenotype-driven diagnosis. Rapid advances in technology and ever decreasing costs have made high-resolution genomic arrays and next-generation sequencing practical for use in a hospital setting. The application of array-comparative genomic hybridization and single-nucleotide polymorphism (SNP) genotyping methods have been used successfully to dramatically increase rates of genetic diagnoses for many disorders including severe developmental delay (3) and intellectual disability (4). Next-generation sequencing methods, particularly exome sequencing, have accelerated the pace of disease diagnosis and gene discovery (5) and are being used in large cohort studies such as the Deciphering Development Disorder project (6).

The rarity and novelty of these disorders, however, mean that the discrimination of a particular variant as being either pathogenic or benign, and correlating this with a specific phenotype, is difficult when viewed in isolation. The large-scale collation and comparison of phenotypes in patients with similar genomic rearrangements, filtered against commonly found variants in the population, aids clinical diagnosis by helping to establish with greater confidence whether the observed variant is likely causal for the phenotype.

The DECIPHER project (DECIPHER–https://decipher.sanger.ac.uk/) was instigated in 2004 to initiate an international collaborative effort to collect and catalogue genotype–phenotype associations, aid the discovery of new syndromes, help in the identification of patients sharing common genotype variants and phenotypes, and highlight common copy-number variants (CNVs) (1,7). As the first database and web-accessible service of its kind, DECIPHER has led the way in the identification of new syndromes (8–14) by encouraging contact and collaboration between participating centres. At the time of writing, DECIPHER contains genotype-linked phenotype data for >21 000 patients deposited from academic clinical genetic centres from 30 countries. DECIPHER provides a secure web interface to its registered users to deposit anonymized patient data (phenotype and genotype) and carry out comparative analysis of these against all data consented for public release (∼9000 patients/∼15 000 variants). With informed patient consent, anonymized linked genotype–phenotype data are made available to all users via the DECIPHER Web site, selected genome browsers (15,16) or as a bulk dataset for research under signed data access agreements. In addition to a powerful search functionality, DECIPHER provides a suite of integrated analysis and visualization tools designed to aid in the interpretation of CNVs and sequence variants.

The overall aims of DECIPHER are as follows:

1. Aid in the interpretation of plausibly pathogenic variants from genome-wide analyses by placing them in the context of known pathogenic variants, other plausibly pathogenic variants and population variation.
2. Annotate plausibly pathogenic variants with their likely functional impact using Ensembl tools to compare sequence and structural variants with the latest functional annotation of the current human reference genome, e.g. define which genes are involved in a specific CNV (microdeletion/microduplication), or for sequence variants, whether they are positioned within a gene or regulatory element.
3. Facilitate research into the study of genes that affect human health and development to improve diagnosis, management and therapy of rare diseases.

DECIPHER achieves these aims by:

- Providing a password-secured confidential repository for the deposition of patient’s genetic findings with accompanying phenotypes.
- Enabling the interpretation of uploaded or queried variant data by providing graphical and tabular views of annotated variant(s) in a patient alongside potentially causal variants seen in other patients.
- Highlighting common phenotypes between deposited patients and those in overlapping positions.
- Displaying expert curated deletion and duplication syndromes that overlap with deposited patient variant data.
- Listing and displaying genes, their genomic positions and scientifically curated information drawn from public biomedical databases, affected by the sequence variant, deletion or duplication.
- Facilitating both inter- and intra-DECIPHER centre collaboration to further the identification of new syndromes, and discovery of genes critical in specific disorders.
- Disseminating public DECIPHER data via a search interface, public genome browsers (UCSC and Ensembl) and through bulk data access for research to improve interpretation of these variants.

**Figure 1.**
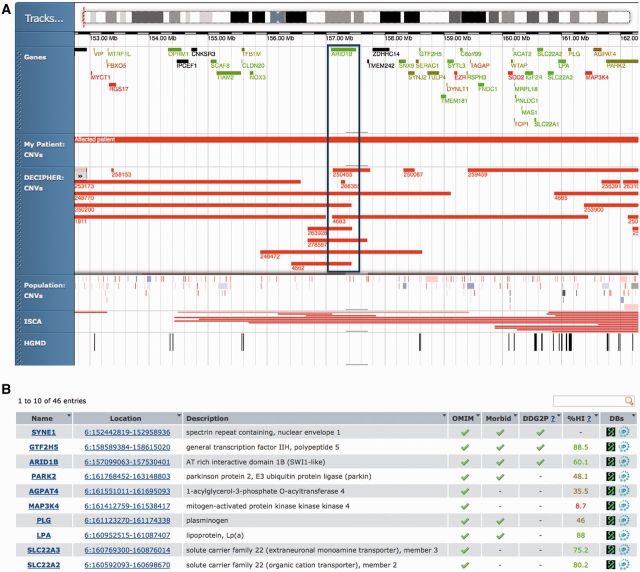

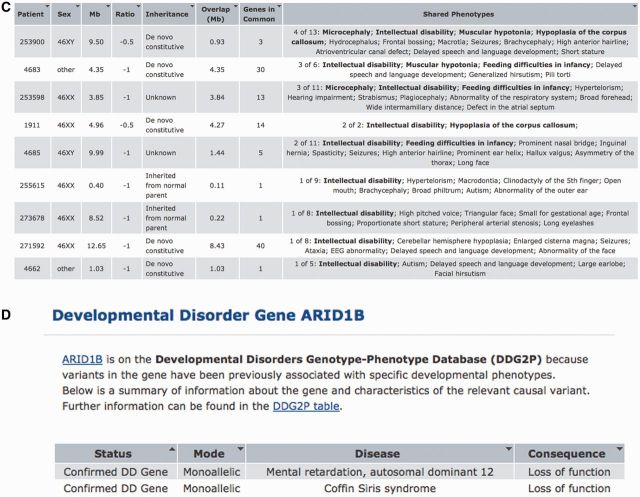
Discovering clusters of patients with a 6q25.3 deletion involved with intellectual disability. Search in DECIPHER for 6q25.3 identified patient 250481 with a 9.5-Mb deletion in 6q25.3. (**A**) Interactive visualization of genes overlapping deletion in patient 250481 and other patients that have similar deletions in 6q25.3 in DECIPHER and ISCA (17) databases. Overlapping genes are coloured by their propensity to display haploinsufficiency (18). (**B**) Prioritizing genes affected by a deletion event in patient 250481. List of all affected genes with associated properties. At least three genes have OMIM Morbid entries and are present in the curated list of genes known to be involved in developmental disorders (DDG2P). (**C**) Identifying other patients with shared phenotypes. Details of overlapping patients with shared phenotypes are highlighted in bold. Most patients appear to display intellectual disability and have a common overlap (see boxed area in Figure 1A) on *ARID1B*. (**D**) Gene *ARID1B* entry in the DDG2P database. Deletions affecting this gene are a cause of intellectual disability.

Figure 1 shows significant features in DECIPHER that allow the discovery of clusters of patients with similar genetic variation and shared phenotypes, thereby driving identification of potentially pathogenic genes or new syndromes.

This article describes critical updates to DECIPHER in 2013 including the development of combined sequence and CNV deposition with associated analysis tools, real-time annotation of deposited sequence variants, deployment of a bespoke interactive genome browser to facilitate combined analysis of sequence and copy-number variation, adoption of the human phenotype ontology (HPO) (19) for phenotype entry and display, as well as other miscellaneous improvements to DECIPHER.

## NEW AND ONGOING DEVELOPMENTS

### Sequence variant deposition

Genetic abnormalities in the genomes of affected patients span a wide spectrum ranging from single nucleotide changes that affect the regulation, expression or activity of a gene to larger deletions or duplications that affect one or more genes. In 2004, when the DECIPHER project was initiated, only large CNVs (varying in size from hundreds of kilobases to several megabases) could be identified genome-wide. DECIPHER has adapted to display smaller and smaller variants as the resolution of genomic array technology has improved, but until now has remained a database of phenotype-linked genotype data coming predominantly from array findings. By extending DECIPHER to encompass most scales and forms of genomic variation, we have created the first utility for the deposition and analysis of both phenotype-linked sequence and copy-number variation. This significant upgrade to DECIPHER functionality creates a portal that will help in the cataloguing of the most common forms of pathogenic variation and/or variants of uncertain significance with associated phenotypes on a per patient basis. Complex double-hit genetic disorders like the thrombocytopenia-absent radius syndrome (20) caused by a combination of copy-number and pathogenic sequence variation can also be easily recorded and analysed in DECIPHER. We have also considered more complex modes of inheritance of variants that are often found in patients with rare disorders and made possible the recording of these using a combination of variant inheritance, parental affected status and phenotypic similarities.

As part of this update, we have also developed back-end storage and user interfaces for the deposition of sequence variation data. The sequence deposition process ensures data accuracy using validation against genomic references (position, reference allele and transcript) before saving to the database. Annotation of the deposited sequence variant is carried out instantaneously and presented to the depositor for further analysis and comparison.

### Sequence and copy-number analysis

**Figure 2.**
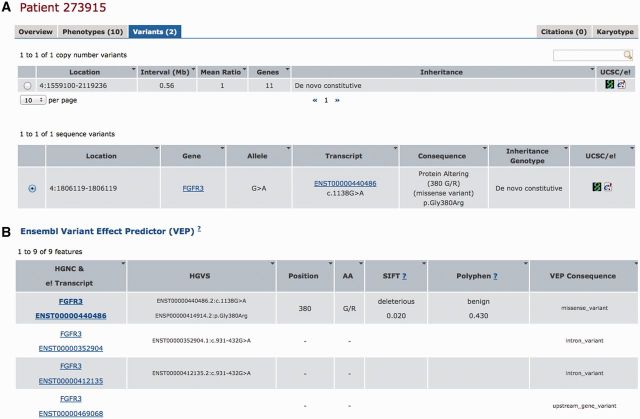
Combined sequence and CNV analysis (e.g. DECIPHER patient 273915). (**A**) Both CNV and sequence variants are shown in tabular form. The sequence variant summary information includes calculated consequences and transcript-based HGVS nomenclature. (**B**) Data from Ensembl VEP for all possible transcripts at given genomic position, with predicted consequences using SIFT (21) and PolyPhen (22). Transcript chosen by the depositor is shown in bold.

To facilitate the interpretation of deposited sequence variants in DECIPHER, we have extended the DECIPHER patient variant pages (see https://decipher.sanger.ac.uk/patient/273915) (Figure 2A) to include derived information from both CNV and sequence variation. The updated pages include a brief summary of the deposited variant with data imported from the Ensembl Variant Effect Predictor (VEP) (23). These include affected gene/s, location (coding, upstream, regulatory regions, etc.) and consequence of the variant (missense, nonsense, stop gain/loss, frameshift, etc.). We have also integrated information from various bioinformatics sources for the affected gene with outgoing links to relevant clinical, genomic or protein resources. An additional tab provides all predictions from Ensembl VEP for the deposited location (Figure 2B). This information contains, among others, predicted consequences using Sequence Ontology (24) terms as well as PolyPhen (22) and SIFT (21) predictions for all known transcripts with associated Human Genome Variation Society (HGVS) (25) identifiers.

### Interactive genome browser

**Figure 3.**
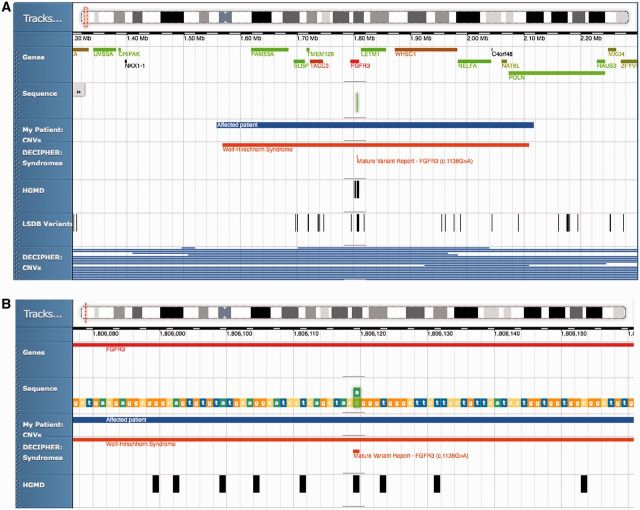
Interactive display of variants in DECIPHER. (**A**) Genome browser showing both sequence and copy-number variation. Data from LSDB (26) and public HGMD (27) databases of sequence variation are also shown with DECIPHER syndrome entries that match these positions. (**B**) At higher zoom levels, the sequence variant is shown on the reference genome sequence. This view shows the G>A sequence variant with overlapping entries in DECIPHER syndromes and HGMD.

We have incorporated a powerful, customizable and interactive genome browser to enhance the utility of DECIPHER (Figures 1A and 3A). The interactive browser (Genoverse: http://www.genoverse.org) has been developed jointly by DECIPHER and Ensembl and is based on the popular HTML5 Canvas technology. The genome browser allows the simultaneous integration of genomic data from multiple sources while facilitating fast and interactive data inspection.

The browser provides a flexible and powerful tool for the analysis of both sequence and CNV data within a common interface. Relevant data are displayed in tracks that may be rearranged, turned on or off or filtered using track-specific controls. Individual items within a track have context-dependent information that provides information and outgoing links to the relevant data source. Tracks may be duplicated using the ‘Tracks’ menu in the browser to visualize data using different filters. Both sequence and CNVs for a single patient are available in a single view along with other patients with overlapping CNVs or sequence variants. Sequence variants for a patient are shown as vertical bars when zoomed out but seamlessly transform to show the variant on the reference genome sequence at high zoom levels (Figure 3B).

The genome browser in DECIPHER integrates data from various pathogenic and population sequence variation and CNV resources to facilitate delineation of the deposited variant. The tracks in the genome browser can be categorized into the following broad themes:

- *Reference Tracks*: These include a scrollable karyotype of the chromosome, a genes track showing all protein-coding genes, a genome sequence track that shows the genomic reference sequence at high zoom levels and any patient sequence variants.
- *DECIPHER Data Tracks*
  - My Patient CNVs: Displays patient CNV as a bar in red (loss) or blue (gain).
  - DECIPHER CNVs: All DECIPHER deposited and consented CNVs that overlap with the patient CNV, filtered by default to show only patients with similar aberrations (gain or loss) and links to specific patient pages. This is modifiable from the track-specific menu.
  - DECIPHER SNVs, InDels: All DECIPHER deposited and consented sequence variants in the region of the patient sequence variant.
  - Research Data: Track of sequence variants from selected high-quality published research studies to aid interpretation of sequence variants.
- *Pathogenic Data Tracks*
  - Syndromes: DECIPHER expert curated syndromes that overlap a given sequence or CNV with links to DECIPHER syndrome pages.
  - ISCA: CNV data from the International Standards for Cytogenomic Arrays (ISCA) (17), filtered by nature of patient CNV (gain or loss) with outgoing links to ISCA for each variant.
  - LSDB variants: All publicly shared locus-specific database (LSDB) (28) sequence variation data from the Leiden Open Variation Databases (26) with outgoing links to actual data in the LSDB.
  - HGMD: All publicly available pathogenic sequence variation data from the Human Genetic Mutation Database (HGMD) (27) with links to data in HGMD.
- *Population Data Tracks*:
  - CNVs: Curated set of common CNV known to occur in the population.
  - SNVs, InDels: Commonly found sequence variants in the population from Ensembl-curated dbSNP, with links to Ensembl.

### Phenotype entry interface

DECIPHER phenotypes were originally based on the commercially available Baraitser–Winter Neurogenetics Database (29). We have now remapped all existing phenotypes in DECIPHER to follow the HPO (19), a comprehensive ontology representing standardized phenotypic terms encountered in humans. We have also introduced an easy ‘drag-and-drop’ interface to enable depositors to rapidly phenotype patients with terms from the HPO vocabulary. As a result, DECIPHER phenotype data are now inter-operable with other resources that use or map to HPO such as ClinVar (http://www.ncbi.nlm.nih.gov/clinvar/) and the ISCA database (17). For those wanting further details about a particular phenotype in a patient, we have added links to the relevant ontology term on the HPO Web site.

### Research data

Academic centres of clinical genetics associated with hospitals have provided all the patient variant data currently held in DECIPHER. The past 5 years of experience of interpreting genome-wide variation has shown the value of combining clinical and research datasets. We have extended the scope of DECIPHER by accepting memberships from academic research groups with expertise in the genomic analysis of rare disease as well as from clinical academic centres for the deposition of phenotype-linked variant data. We believe that this will increase coverage of the variation spectrum in DECIPHER, while at the same time engaging the research and clinical communities in the identification of patient clusters with similar findings.

### Data-sharing

The anonymous consented patient data in DECIPHER contain a wealth of phenotype-associated genotype data, ideal for identifying novel disease genes, association studies, research or the development and/or validation of new bioinformatics tools. DECIPHER makes all consented anonymous data available as a frequently updated encrypted download for all *bona fide* academic researchers under a formal data access agreement. The use of these data for visualization purposes is covered by a separate Data Display Agreement, both of which may be found on the DECIPHER Web site. The anonymous bulk data are available in multiple formats for use in research or in-house display.

Examples of publications derived from DECIPHER bulk data interrogation include the following:

- Prediction of haploinsufficiency: Huang *et al.*(18) used DECIPHER data in their predictions on the propensity of a gene to demonstrate haploinsufficiency, a measure of the ability of a single copy of a gene to maintain normal function.
- Mouse–human phenotype association studies: This study by Boulding and Webber (30) associated critical human genes with clinical symptoms by disruption of mouse orthologous genes whose disruption produces similar murine phenotypes.
- Studies on copy number-stable regions: Johansson and Feuk (31) have described the correlation between copy number-stable regions of the human genome and rare inherited variants using clinical data from DECIPHER and other sources.

## SUMMARY AND OUTLOOK

DECIPHER is an invaluable scientific and clinical resource for clinical genetics and research. The database has grown from ∼2000 patients in 100 centres in 2009 to >21 000 patients (∼9000 consented) in >200 centres in 2013 (up-to-date statistics are available from the Web site). The benefits of increased data sharing through patient consent has in turn led to an increase in the number of international collaborations between depositing clinical genetic centres of patients with shared genotype–phenotype features. The results of these efforts have accelerated the rate of discovery of new syndromes and delineation of critical genes contributing to their causation: in 2012, DECIPHER data were cited in >160 peer-reviewed publications. DECIPHER is now accessed regularly by >10 000 unique visitors every month from >90 countries worldwide, and has become a standard tool in routine clinical interpretation of genomic variants. We believe that accepting sequence variants in addition to CNVs further enhances the utility of DECIPHER and provides a facility for analysing the whole spectrum of genomic variation with associated phenotypes and inheritance in a single place. The inclusion of research data in DECIPHER will catalyze interactions between clinical and research communities leading to more rapid and accurate interpretation of plausibly pathogenic variation.

DECIPHER currently has two tiers of data accessibility: (i) only visible within a single centre, or (ii) visible to the entire world, subject to explicit consent. To facilitate greater sharing of data, we are working on the introduction of additional, intermediate levels of data access where deposited data could be made available to selected members of a collaborative project or all registered users of DECIPHER. We are also working on the introduction of an automated notification system that will inform depositors when a similar variant is consented for sharing, facilitating reinterpretation of previously deposited variants and the collaborative discovery of novel genetic diseases.

## CONTACTING US

DECIPHER has a dedicated helpdesk email address: decipher@sanger.ac.uk for feedback, information or bug reports. In addition, we run a mailing list (http://publists.sanger.ac.uk/mailman/listinfo/decipher-announce) to which we send important news and notifications. We are open and receptive to ideas and suggestions about how to use DECIPHER to improve the interpretation of plausibly pathogenic variants.

## FUNDING

The Wellcome Trust [WT077008]. Funding for open access charge: Wellcome Trust Sanger Institute Funding.

*Conflict of interest statement*. None declared.
